# Effects of a second iron-dextran injection administered to piglets during lactation on differential gene expression in liver and duodenum at weaning

**DOI:** 10.1093/jas/skae005

**Published:** 2024-01-14

**Authors:** James L Pierce, J Wesley Lyons, Tyler B Chevalier, Merlin D Lindemann

**Affiliations:** James Pierce Consulting, Nicholasville, KY 40356, USA; Department of Animal and Food Sciences, University of Kentucky, Lexington, KY 40506, USA; Pharmacosmos Inc., Watchung, NJ 07069, USA; Department of Animal and Food Sciences, University of Kentucky, Lexington, KY 40506, USA; Department of Animal and Food Sciences, University of Kentucky, Lexington, KY 40506, USA

**Keywords:** gene expression, iron, iron dextran, iron deficiency anemia, piglet, RNA-Seq

## Abstract

Six female littermate piglets were used in an experiment to evaluate the mRNA expression in tissues from piglets given one or two 1 mL injections of iron dextran (200 mg Fe/mL). All piglets in the litter were administered the first 1 mL injection < 24 h after birth. On day 7, piglets were paired by weight (mean body weight = 1.72 ± 0.13 kg) and one piglet from each pair was randomly selected as control (CON) and the other received a second injection (+Fe). At weaning on day 22, each piglet was anesthetized, and samples of liver and duodenum were taken from the anesthetized piglets and preserved until mRNA extraction. differential gene expression data were analyzed with a fold change cutoff (FC) of |1.2| *P *< 0.05. Pathway analysis was conducted with Z-score cutoff of *P *< 0.05. In the duodenum 435 genes were significantly changed with a FC ≥ |1.2| *P* < 0.05. In the duodenum, Claudin 1 and Claudin 2 were inversely affected by + Fe. Claudin 1 (CLDN1) plays a key role in cell-to-cell adhesion in the epithelial cell sheets and was upregulated (FC = 4.48, *P* = 0.0423). Claudin 2 (CLDN2) is expressed in cation leaky epithelia, especially during disease or inflammation and was downregulated (FC = −1.41, *P* = 0.0097). In the liver, 362 genes were expressed with a FC ≥ |1.2| *P* < 0.05. The gene most affected by a second dose of 200 mg Fe was hepcidin antimicrobial peptide (HAMP) with a FC of 40.8. HAMP is a liver-produced hormone that is the main circulating regulator of Fe absorption and distribution across tissues. It also controls the major flows of Fe into plasma by promoting endocytosis and degradation of ferroportin (SLC4A1). This leads to the retention of Fe in Fe-exporting cells and decreased flow of Fe into plasma. Gene expression related to metabolic pathway changes in the duodenum and liver provides evidence for the improved feed conversion and growth rates in piglets given two iron injections preweaning with contemporary pigs in a companion study. In the duodenum, there is a downregulation of gene clusters associated with gluconeogenesis (*P* < 0.05). Concurrently, there was a decrease in the mRNA expression of genes for enzymes required for urea production in the liver (*P* < 0.05). These observations suggest that there may be less need for gluconeogenesis, and possibly less urea production from deaminated amino acids. The genomic and pathway analyses provided empirical evidence linking gene expression with phenotypic observations of piglet health and growth improvements.

## Teaser

1. Pigs that received two injections of iron-dextran before 2 wk of age were numerically heavier and exhibited greater blood Hb concentrations at weaning.

2. Administering a second iron injection resulted in changes in the expression of 362 genes in the liver and 435 genes expressed in small intestine.

## Introduction

Increased litter size combined with faster-growing piglets that are consuming an all-milk diet increases the risk of developing subclinical or full-scale iron deficiency anemia (IDA) (Gaddy, Prussman, and Gable, 2012). An administration of 200 mg iron at 1 to 3 d of age has been demonstrated to meet the iron need of piglets until only ~4 kg body weight (BW) due to the dilution of iron stores with body growth from birth and low levels of iron in sow milk (~1 mg daily intake). The combination of larger litters of faster-growing piglets can contribute to a potential “iron gap” before weaning (Van Gorp, Segers, and von der Recke, 2012) Iron deficient anemia has been studied extensively since production systems moved indoors.

The study of gene expression related to iron status in piglets has steadily progressed in recent years. However, in the past, individual genes of interest were first selected, and then the expression was determined with quantitative real-time polymerase chain reaction (qRT-PCR) and microarray analysis of messenger RNA (mRNA). Currently, the technique of RNA sequencing (RNA-seq) is an extremely robust method available for evaluating the expression of genes across the entire genome (Finotello and Di Camillo, 2015). It is currently the most powerful tool for the study of differential gene expression (DGE) with high precision reducing the need for replicates typically needed for evaluating biological samples (Conesa et al., 2016). The hypothesis was that several genes related to iron metabolism in the tissues evaluated would be demonstrated to be either up- or downregulated and that the robust RNA sequencing method would result in the discovery of unanticipated changes in gene expression related to aspects of metabolism that have not been associated with iron status to date. These unanticipated changes provide opportunities for more detailed experimentations related to iron status in the future.

Therefore, the objective of this experiment was to evaluate the DGE at weaning between piglets receiving one or two 200 mg Fe dextran injections by RNA Sequencing (RNA-Seq) of liver and duodenal samples.

## Materials and Methods

The experiment was conducted in an environmentally controlled room at the University of Kentucky Swine Research Center. The experiment was conducted under protocols approved by the Institutional Animal Care and Use Committee of the University of Kentucky.

### Animals and experimental design

Six female littermate pairs from a single letter (Yorkshire × Landrace × Large White; initial BW 1.72 ± 0.13 kg) were used in an experiment to evaluate the DEG. The sow was fed a corn–soybean meal diet supplemented with 110 ppm Fe as FeSO_4_ as described by Chevalier et al. (2023). The piglets did not have access to creep feed. All piglets in the litter were administered 1 mL of iron dextran (200 mg Fe/mL, Uniferon, Pharmacosmos Inc. Watchung, NJ) by an intramuscular (IM) injection < 24 h after birth. On day 7, piglets were paired by weight (BW difference of < 0.23 kg), and one piglet from each pair was randomly selected as the control (CON) while the other received a second 1 mL injection (+Fe).

### Measurements and sample collection

Hemoglobin concentration (Hb) was measured at birth, day 7, and weaning using a HemoCue Hb 201 + analyzer (HemoCue America, Brea, California). The HemoCue Hb 201 + was previously validated to assess Hb in arterial blood of piglets (Kutter et al., 2012). Blood samples were taken from the ear veins of the piglets before the initial and second iron injections were administered and loaded into disposable microcuvettes via capillary action. The microcuvette was placed in the HemoCue Hb 201 + and the resulting Hb concentration was displayed and recorded within 60 s. BW was recorded at birth, day 7, and weaning to determine average daily gain (ADG).

At weaning on day 22 (mean BW = 4.92 ± 0.45 kg) each piglet was anesthetized via IM injection of a 50:50 mixture of Xylamed (Xylazine 100 mg/mL) and ketamine HCl (100 mg/mL) to reconstitute a 5 mL bottle of Telazol (Zoetis, Kalamazoo, MI). Piglets were dosed at a rate of 1 mL/50 pounds (~0.3 mL/piglet). Samples of liver and duodenum were taken from the anesthetized piglet and placed in DNA/RNA Shield (Zymo Research, Irvine, CA) for preservation until mRNA extraction.

### Statistical analysis of growth, tissue weight, and hemoglobin

Data analyses were performed in SAS 9.4 (SAS Inst. Inc., Cary, NC, USA) by least-squares analysis of variance using the generalized linear model as a randomized complete block design. The individual piglet served as the experimental unit for BW, ADG, Hb, and tissue weight data. The statistical model included terms for treatment and pair. Statistically significant differences were established at *P* ≤ 0.05; tendencies were established at *P* ≤ 0.10 for main effects.

### RNA sequencing

Samples were submitted to Zymo Research (Irvine, CA, USA) for total mRNA extraction, cDNA library preparation, and RNA sequencing. Total RNA-Seq libraries for pigs were constructed from 250 ng of total RNA. To remove rRNA, a method described by Bogdanova et al., 2011 was followed. Libraries were prepared using the Zymo-Seq RiboFree Total RNA Library Prep Kit (Cat # R3000) according to the manufacturer’s instructions (Zymo-Research, 2022). The RNA-Seq libraries were sequenced on an Illumina NovaSeq to a sequencing depth of at least 30 million read pairs (150 bp paired-end sequencing) per sample.

### Detection of DGE and bioinformatic data handling

Differentially expressed genes were detected using GeneSpring software (Agilent, Santa Clara, CA) using selection criteria that accepted a DGE threshold of greater than a |1.2|-FC in expression level and statistical probability levels of *P* < 0.05.

The filtered genes were then subjected to Ingenuity Pathway Analysis (QIAGEN Inc., Redwood City, CA; https://digitalinsights.qiagen.com/products/) to gain insights into canonical pathways, networks, and biological functions. Qiagen Ingenuity Pathway Analysis uses algorithms, tools, and visualizations to combine the directional information from gene expression patterns (up- or down-regulation) with the expected causal effects of the genes, as reported in the published literature. A prediction for effects of a treatment on a particular biological pathway function or disease can then be made based on the direction of change in gene expression and calculated Z-scores. Briefly, a Z-score is used to compare data that have different means and standard deviations. The Z-score is the distance of a point, such as a complete pathway, from the mean of the distribution in terms of the standard deviation (Corchete et al., 2020; Shao et al., 2020; Wieder, Lai and Ebbels, 2022).

## Results and Discussion

### Growth performance, hemoglobin, and tissue weight

Not surprisingly, there were no significant changes in BW, Hb, or tissue weights (Table 1) given that the experiment was designed for the determination of DGE with only three pairs of piglets (a number inadequate for the statistical power needed to reach a significance of *P* < 0.05 for performance response measures). However, there were expected numerical improvements in both BW and Hb at weaning observed in piglets that received two injections. The mean Hb concentration in the CON piglets was 10.5 g/dL, which is lower than a reported lower critical limit of ~11.0 g/dL (Bhattarai and Nielsen, 2015) suggesting these piglets may have been subclinically deficient in iron compared with 12.7 g/dL Hb in the + Fe piglets. In a study with contemporary pigs from these littermates that were obtained from pigs in the same farrowing room, treated with a second iron injection on the same day, and weaned at the same time (Chevalier et al., 2023) the Hb values for the two groups at weaning were 10.7 and 13.1 g/dL (*P* < 0.01) and the pigs had greater ADG from weaning to slaughter (0.81 vs. 0.83 kg/d; *P* = 0.04). Liver (a target tissue for gene expression responses herein) weights were numerically lower on an absolute (126.0 g vs. 123.2 g) and relative basis (percent of BW; 2.64 vs. 2.47%) for the + Fe pigs. Conversely, absolute spleen (an organ playing important roles related to hematology and immunology) weights increased numerically in piglets receiving two injections (11.8 g vs. 14.7 g) and as a percent of BW (0.26% vs. 0.29%).

**Table 1.** Summary of body weight (BW), hemoglobin (Hb) at birth, second injection (day 7), and weaning (day 22)^1^

	TRT			
Variables	CON	+Fe	SEM	*P*-value
*n*	3	3		
BW, kg				
Birth	1.15	1.23	0.09	0.58
Second injection	1.70	1.74	0.03	0.47
Weaning	4.84	5.00	0.14	0.52
Hb, g/dL				
Birth	10.4	10.3	0.85	0.96
Second injection	9.5	9.5	0.44	0.96
Weaning	10.5	12.7	0.74	0.17
Tissue weight, g				
Liver	126.0	123.2	2.52	0.52
Spleen	11.8	14.7	1.30	0.25
Heart	28.7	29.7	0.94	0.53
Kidneys^2^	28.6	28.0	0.75	0.63
Tissue weight, % of BW				
Liver	2.64	2.47	0.08	0.28
Spleen	0.26	0.29	0.03	0.54
Heart	0.61	0.60	0.02	0.87
Kidneys^2^	0.60	0.56	0.03	0.40

^1^All piglets were administered a single iron-dextran injection (200 mg Fe) at birth (< 24 h); the second iron injection group received a second iron injection (200 mg Fe) on day 7. Pigs were weaned at 22 d.

^2^Both kidneys were harvested at weaning on day 22.

Tissue weights were taken at weaning (day 22).

Because more than 27,000 genes were assessed in each tissue by the sequencing methods used, it is impossible to present all results, or even just the significant results, in a single publication. Therefore, selected results of primary interest to the authors are presented herein and the entirety of significant changes in gene expression data set made available to interested parties at *Journal of Animal Science.*

### Duodenum DGE

**Figure 1.**
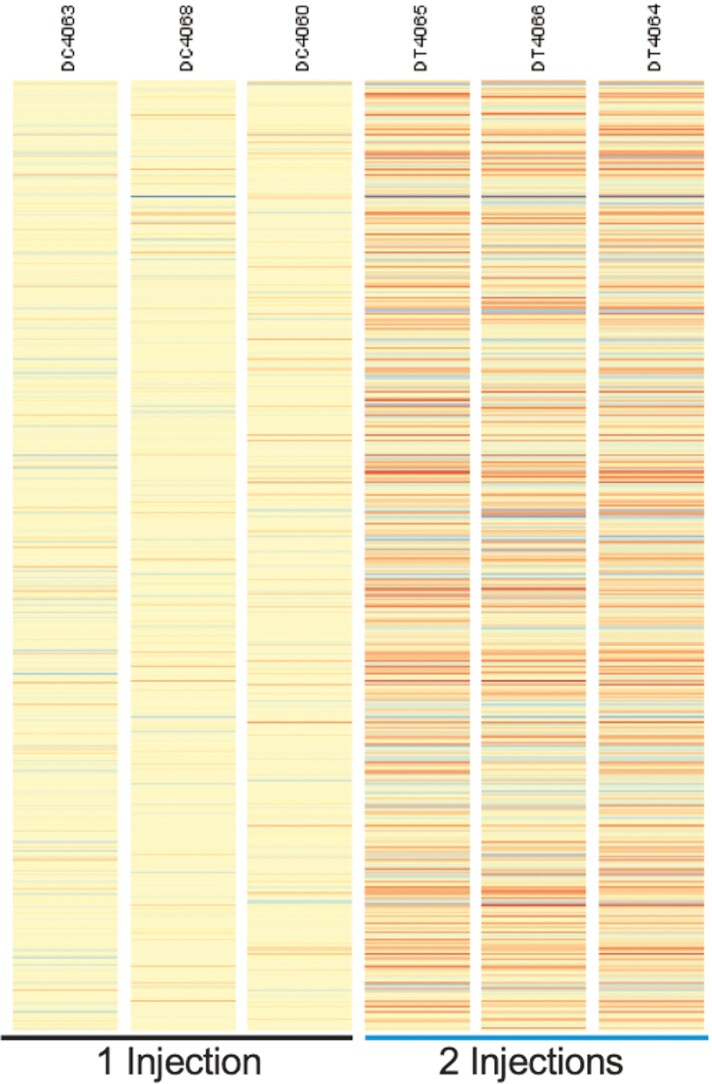
Heat map of differentially expressed genes in the duodenum due to number of iron injections (*P* < 0.05). Each column on the heatmap represents an individual piglet (*n* = 3/injection treatment). Every line within each column represents one of the 435 genes that were expressed differently >|1.2| (*P* < 0.05). Orange is used to convey predicted increases in a gene expression while blue is used to indicate decreases in gene expression. Intensity of the color is the relative magnitude of response.

**Figure 2.**
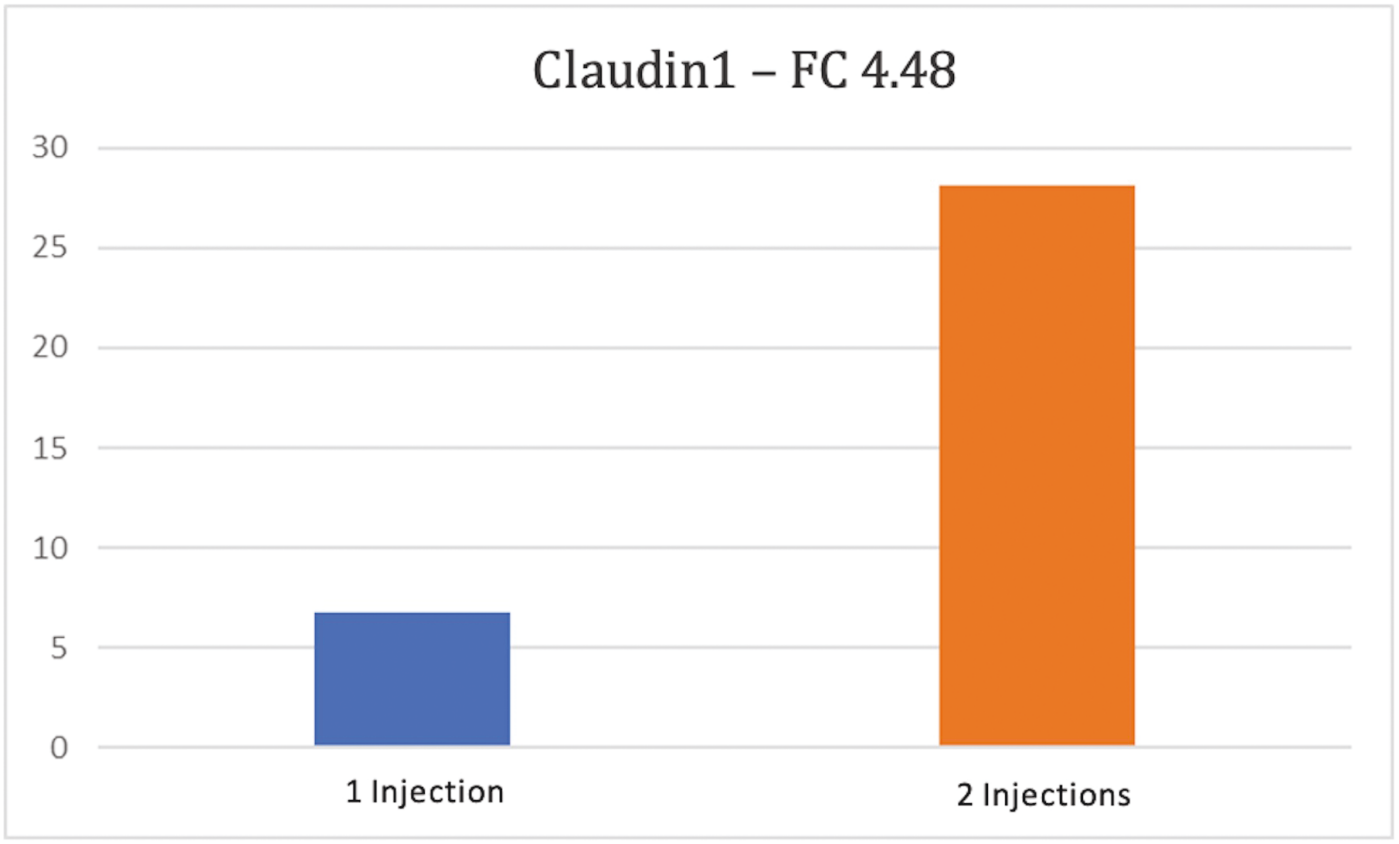
Claudin1 gene expression in the duodenum of piglets receiving 1 or 2 iron injections (*P* < 0.05). Piglets that received a second dose of 200 mg iron dextran on day seven exhibited a 4.48 FC increase in Claudin1 gene expression (*P* < 0.05). Claudin1 is highly involved in tight junctions between epithelial cells (*n* = 3/injection treatment).

In the duodenum, 435 genes were significantly changed with a fold change (FC) ≥ |1.2| *P* < 0.05 (Figure 1). Most notably, CLDN1 and CLDN2 which code for the transmembrane proteins known as Claudin 1 and Claudin 2, respectively, were inversely affected by the second dose of iron (Table 2). These findings support the expected interaction between the two proteins (Markov, Aschenbach, and Amasheh, 2015). The CLDN1 gene (Figure 2, FC = 4.48, *P* = 0.0423) was upregulated and is critical for cell-to-cell adhesion in the epithelial cell sheets and serves as a barrier to prevent water, bacteria, and solutes from passing through the paracellular space (France and Turner, 2017). In contrast, CLDN2 (FC = −1.41, *P* = 0.0097) expression was downregulated which has been shown to be associated with cation leaky epithelia (France and Turner, 2017; Suzuki, 2020). Interestingly, because the claudin-2 protein provides a paracellular channel for water and cations, higher expression of CLDN2 has been observed in diseased intestines (Luettig et al., 2015).

**Table 2.** Fold change (FC) expression of select genes in the duodenum at weaning in response to a second iron injection in piglets^1^

Gene symbol	FC	*P*-value	Gene name	Gene ID	Associated biological functions
CLDN1	4.48	0.0424	Claudin 1	100625166	Tight junction formation between epithelial cells
CLDN2	−1.41	0.0097	Claudin 2	733684	Leaky junction and paracellular transport between epithelial cells
TFRC1	−2.89	0.0001	Transferrin receptor protein 1	397062	Receptor-mediated endocytosis of transferrin
HFE	−1.33	0.0386	Homeostatic iron regulator	110261476	Influences Fe absorption by regulating HAMP and the transferrin receptor with transferrin
GPX-3	−1.69	0.0225	Glutathione peroxidase 3	396598	Selenium-containing enzyme that scavenges reactive oxygen species
PCSK9	−2.76	0.0065	Proprotein convertase subtillisin/kexin type 9	100620501	Contributes to cholesterol homeostasis of enterocytes
SREBF2	−1.25	0.0315	Sterol regulatory element binding transcription factor 2	396675	Acts as central hub in regulation of cell lipid levels

^1^Postive and negative FC represent up or down regulation, respectively.

There was a −2.89 FC reduction (*P* = 0.001) in the expression of TFRC1, which is critical for receptor-mediated endocytosis of transferrin. Because the + Fe piglets had a favorable iron status based on Hb concentration at weaning (Table 1) it is logical that there would be less TFRC1 expressed compared with the CON piglets. These results agree with results from experiments with high-iron diets fed to piglets (Hansen et al., 2010) and rats (F. Dupric et al., 2002).

In the current experiment, the homeostatic iron regulator gene (HFE) was downregulated (−1.33 *P* = 0.039) in the duodenum of the + Fe treatment to piglets (Table 2). This is the opposite of results reported from a study with human intestinal (Caco-2) cells in culture (Han et al., 1999). The authors reported that HFE expression increased by increasing cellular iron uptake in human intestinal cells. These differences observed between studies are likely due to the present experiment administering an IM injection of iron dextran thereby bypassing dietary iron being absorbed from the intestinal lumen.

Another interesting observation was the decreased expression of glutathione peroxidase three (GPx-3, Table 2) in the + Fe piglets (−1.69 FC, *P* = 0.023). GPx-3 is produced in the kidney but is the only extracellular member of the glutathione peroxidase family (Burk et al., 2011). Because hypoxia leads to an increased formation of reactive oxygen species from the mitochondria, the prevention of hypoxia by the + Fe treatment may be responsible for a decreased requirement of GPX-3, as well as dietary Se (Bierl et al., 2004).

### Duodenum pathway analysis

To evaluate the overall outcomes of the effects related to a second iron injection, genes that were differentially expressed in + Fe piglets vs. CON piglets were categorized according to gene ontology with Ingenuity Pathway Analysis software (Table 3). Some of the most surprising and interesting observations of this analysis were the downregulation of metabolic processes involved in gluconeogenesis.

**Table 3.** Examples of biochemical pathway changes as estimated by gene clustering in the duodenum in + Fe piglets compared with CON piglets (*P* < 0.05)^1^

Biochemical pathway	Description	*Z-*score
*Synthesis*		
Gluconeogenesis	Formation of carbohydrates from non-carbohydrates	−1.671
Lipids	The synthesis of lipids from glucose	−0.908
*Metabolism*		
Sterol	Involved in cell membrane maintenance, gene expression, and hormone production.	−1.966
Steroid	Subclass of sterols with a specific side chain. They are produced from cholesterol. Steroid hormones include testosterone, estrogen, and cortisol as examples.	−1.850
Proliferation of epithelial cells	Upregulated genes related to gut health and development.	0.361

^1^A Z-score is used to compare data that have different means and standard deviations. The Z-score is the distance of a point, such as a complete pathway, from the mean of the distribution in terms of the standard deviation as described by Weider et al. (2022).

Iron deficiency, especially IDA, has been demonstrated to induce hyperglycemia (Davis et al., 2012; Soliman et al., 2017; Tangvarasittichai, 2018). However, by applying pathway analysis for the duodenum (Table 3) a reduction in the gene expression associated with biological pathways associated with gluconeogenesis from amino acids (Z = −1.671, *P* < 0.05) and lipid synthesis from glucose was observed (*Z *= −0.908, *P* < 0.05). This agrees with observations in humans and rodents whereby slight iron deficiency alters glucose metabolism (Soliman et al., 2017; Dziegala et al., 2018; Tangvarasittichai, 2018) turnover, and oxidation (Henderson, P. and Brooks, 1986). Gluconeogenesis from lactate requires two ATP, and four ATP from glycerol. The most energetically expensive source of glucose from gluconeogenesis are glucogenic amino acids as precursors which require six ATP per molecule of glucose produced (Murray et al., 2003). While we cannot simply count the ATP used into calories wasted (Melzer, 2011), if the change in gene expression is ultimately demonstrated to be accompanied by a change in the metabolic flux through those pathways, the implication of ATP savings to animal energetic efficiency and whole-body feed efficiency is intuitively obvious.

The gene expression of pathways associated with lipid synthesis was also downregulated (Z-score = −0.908 [*P* < 0.05]). A decrease in lipids, cholesterol, and triglycerides has been reported from a human clinical experiment evaluating normal vs. IDA patients (Shirvani et al., 2017). However, no molecular mechanisms were evaluated in that cross-sectional study. Those observations contrast with results from an experiment with rats (Davis et al., 2012). It should be noted that the iron-deficient rats were severely anemic to the control with Hb concentrations less than half of the control 6.6 vs. 13.5 g/dL, respectively. The piglets in this experiment were not anemic based on comparisons with several research reports (Patience and Gabler, 2012; Bhattarai and Nielsen, 2015; Ventrella et al., 2017; Knight and Dilger, 2018).

Finally, in + Fe piglets there were significant decreases in gene expression of biological pathways associated with sterols involved in cell membrane maintenance and hormone production (*Z*-score = −1.966, *P* < 0.05). Pathways associated with steroid metabolism from cholesterol were also significantly downregulated (*Z*-score = −1.850, *P* < 0.05). However, there was an increase in the genes related to proliferation of epithelial cells (*Z*-score = 0.361, *P* < 0.05) as shown in Table 3.

### Liver DGE

**Figure 3.**
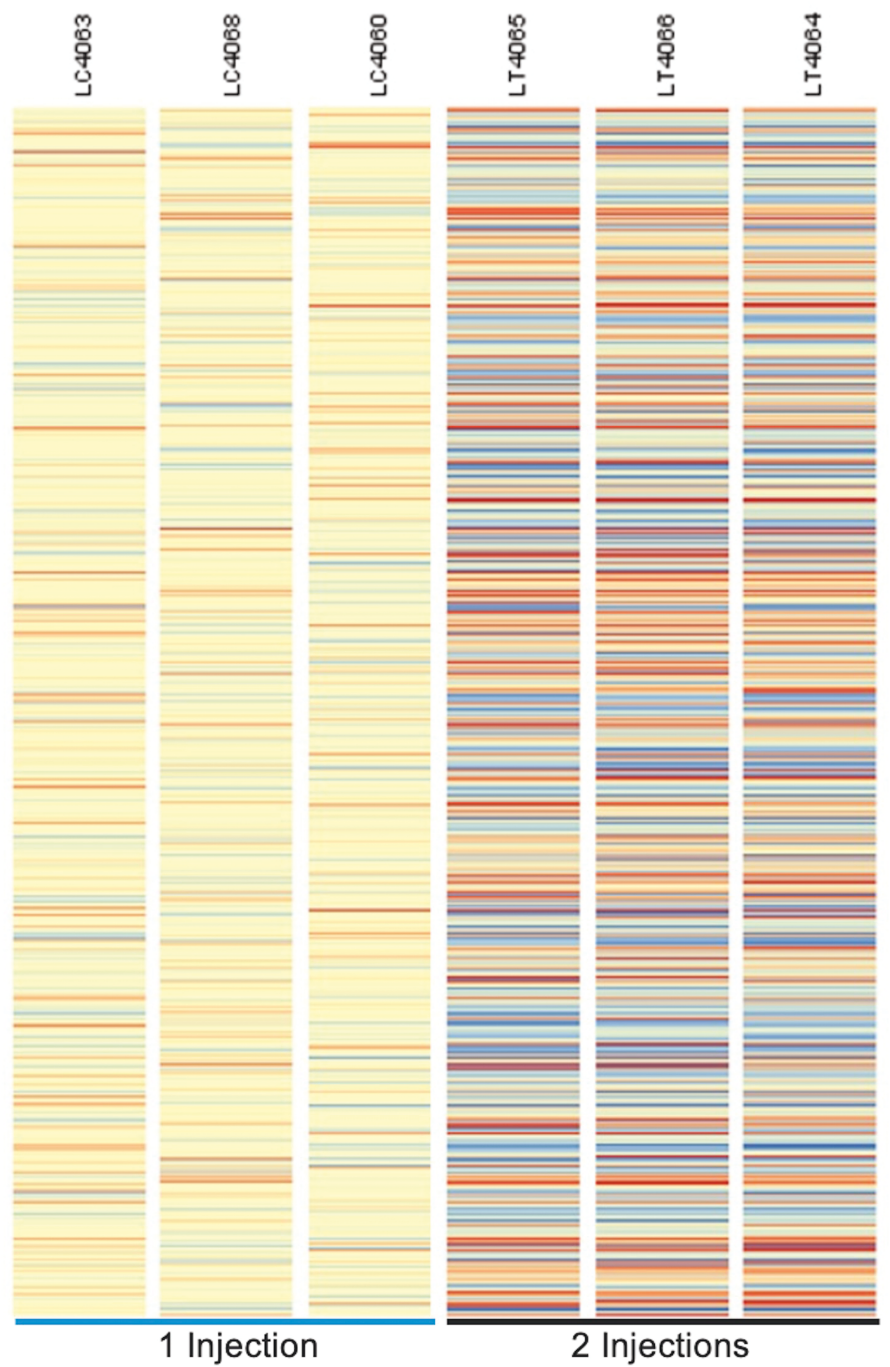
Heat map of differentially expressed genes in liver due to number of iron injections (P < 0.05). Each column on the heatmap represents an individual piglet (*n* = 3/injection treatment). Every line within each column represents one of the 362 genes that were expressed differently in the liver >|1.2| (*P* < 0.05). Orange is used to convey predicted increases in a gene expression while blue is used to indicate decreases in gene expression. Intensity of the color is the relative magnitude of response.

**Figure 4.**
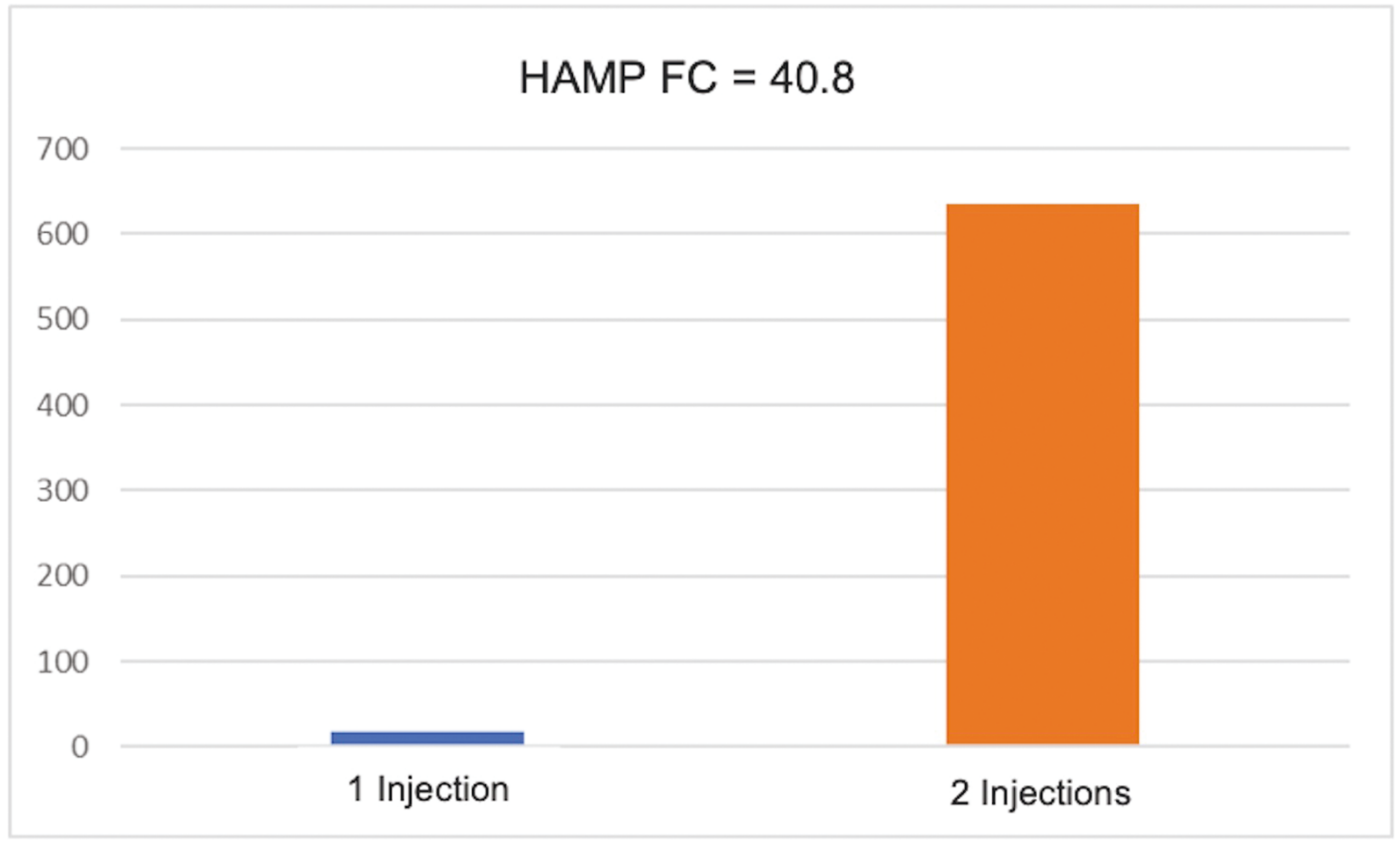
The expression of Hepcidin Antimicrobial Peptide (HAMP) in liver was affected by one or two iron-dextran injections before weaning (*P* < 0.05). Hepcidin (HAMP) regulates plasma iron concentrations by controlling ferroportin concentrations in iron-exporting cells. Hepatocyte hepcidin synthesis is regulated at the transcriptional level by hepatic iron stores, erythropoiesis, hypoxia, infection, and inflammation (*n* = 3/injection treatment).

The heatmap in Figure 3 shows 362 genes from + Fe piglets were differentially expressed with a FC ≥ |1.2| *P* < 0.05. The gene most upregulated in + Fe piglets was hepcidin antimicrobial peptide (HAMP) with a FC of 40.8 (*P* = 0.0073) as shown in Figure 4. Hepcidin antimicrobial peptide is a liver-produced hormone that is the main circulating regulator of Fe absorption and distribution across tissues (Park et al., 2001). It also controls the major flows of Fe into plasma by promoting endocytosis and degradation of ferroportin (SLC4A1). This leads to the retention of Fe in Fe-exporting cells and decreased Fe flow into plasma (Hansen et al., 2010; Bogdan et al., 2016). Also of note is the upregulation of bone morphogenic protein 6 (FC 1.31, *P* = 0.0168). It is a protein that plays a role in several processes such as bone development, wound healing, and Fe metabolism. Bone morphogenic protein 6 modulates iron homeostasis by hepcidin expression at the level of transcription (Rausa et al., 2015; Kowdley et al., 2021).

Thermoregulation of humans, pigs, and rats with IDA results in activation of both the sympathetic nervous system and the pituitary-thyroid axis (Fajt et al., 2016). However, IDA in rats has been attributed almost solely to low Hb or hematocrits resulting in diminished oxygen delivery to tissues for heat production which is a major component of cold sensitivity (Beard et al., 1984). In a study involving 11 women who underwent iron depletion, followed by repletion, the women demonstrated shivering significantly earlier, and had lower skin and core body temperatures during iron deficiency compared with iron repletion (Lukaski, Hall and Nielsen, 1990). In the current experiment, body temperatures were not measured, however; there was a significant FC increase in transient receptor channel potential vanilloid 1 (TRPV1, 4.18, *P* = 0.0132). While most experiments associate TRPV1 with heat, pain, and capsaicin effects on thermoregulation (Gavva et al., 2007; Szolcsanyi, 2015), limited work has been conducted on a definitive link between iron status and thermoregulation (Rossi et al., 2022).

### Liver pathway analysis

Metabolic pathway changes in liver (Table 4) not only supported the improved hematological measures observed with a second iron injection but also provided evidence supporting the improved postweaning growth rate in piglets administered two iron injections postweaning (Chevalier, 2019; Chevalier et al., 2023). As presented in the duodenum, where there was a downregulation of gene clusters involved in gluconeogenesis (*P* < 0.05); concurrently, there was a decrease in the expression of gene clusters associated with the production of urea in the liver (*P* < 0.05). These observations suggest that there may be less need for gluconeogenesis, thus less urea production from deaminated amino acids. This can also lead to a substantial savings in maintenance energy because every molecule of urea produced requires four ATP (Bender, 2012). The DGE and pathway analyses provide evidence linking gene expression with phenotypic observations of piglet health and growth (Knight and Dilger, 2018; Chevalier, 2019; Chevalier et al., 2023).

**Table 4.** Examples of biochemical pathway changes as estimated by gene clustering in the liver (*P* < 0.05) in + Fe piglets compared with CON piglets^1^

Biochemical pathway	Description	*Z score*
*Iron* q*uantity*		
Urea synthesis	Urea synthesized from deaminated amino acids	−0.832
Vitamin D metabolism	Formation of 1,25-dihydroxyvitamin D	1.955
*Hematology*		
Hematocrit	Percentage of red blood cells in whole blood	1.360
Hemostasis	Platelet aggregation and coagulation	0.848
Red blood platelet activation	Increased adhesion to form a clot to prevent bleeding from wounds	0.827

^1^A Z-score is used to compare data that have different means and standard deviations. The Z-score is the distance of a point, such as a complete pathway, from the mean of the distribution in terms of the standard deviation as described by Weider et al. (2022).

Iron is required as a cofactor for the enzyme that converts Vitamin D (cholecalciferol), a prohormone nutrient involved in skeletal development (Azizi-Soleiman et al., 2016). The + Fe piglets were found to have a significantly upregulated expression of genes associated with vitamin D metabolism pathway (Z-score = 1.955, *P* < 0.05) as shown in Table 4. Furthermore, the + Fe piglets also significantly expressed more CYP2R1 (1.29 FC, *P* = 0.0345, Table 5) which codes for the iron-dependent, rate-limiting enzyme which is part of the cytochrome P450 superfamily, which are heme-containing monooxygenases 25-hydroxylase which catalyzes the addition of a hydroxyl group to the 25th carbon of cholecalciferol to form 25-hydroxyvitamin D (Toxqui and Vaquero, 2015; Qiu et al., 2022). Therefore, the requirement of iron in the two-step hydroxylation process by the enzymes coded by CYP2R1 and CYP27A1, respectively, has led some researchers to conclude that appropriate iron supplementation may promote Vitamin D3 production which could result in improved Ca and P absorption as well as proper bone growth and development (Toxqui and Vaquero, 2015; Azizi-Soleiman et al., 2016; Qiu et al., 2022).

**Table 5.** Fold change (FC) in expression of select genes in the liver at weaning in response to a second iron injection in piglets

Gene symbol	FC	*P*-value	Gene name	Gene ID	Associated biological functions
BMP6	1.32	0.0168	Bone morphogenic protein 6	100155536	Regulator of hepcidin expression
CD59	1.25	0.0281	Glycolipid-anchored glycoprotein	397347	Regulates complement-mediated cell lysis, and hemolytic anemia
CYP2R1	1.29	0.0345	Cytochrome P450 family 2 R1	100124375	Encodes 25-hydroxylase. Catalyzes addition of hydroxyl group to the 25th carbon of cholecalciferol.
EGLN3	−1.56	0.0242	Egl-9 hypoxia-inducible factor 3	100152368	Encodes the protein prolyl hydroxylase 3 during hypoxia
EPB42	−2.02	0.0435	Erythrocyte membrane protein band 4.2	100152957	Neutrophils—humoral immune response
F2RL2	−7.38	0.0036	Coagulation factor II receptor-like 2	100519355	A cofactor in thrombin-mediated cleavage and activation of PAR4.
GP6	2.68	0.0203	Glycoprotein VI	100517829	Platelet-specific collagen receptor for blood clots
HAMP	40.8	0.0073	Hepcidin antimicrobial peptide	397207	Hormone regulates iron release from ferroportin
LGALS1	−1.37	0.0275	Galectin 1, beta-galactoside- binding proteins	414915	Modulates cell-cell and cell-matrix interactions
NMUR1	4.4	0.0001	Neuromedin U receptor 1	100520336	Regulates osteoblast differentiation and activity
NUCB2	−1.41	0.0054	Nucleobindin 2	100512826	Neuropeptide serves roles in regulating food intake
SLC11A1	2.06	0.0398	Solute carrier 11-member 1	396764	Transporter for Fe and other divalent cations
SLC4A1	4.70	0.0155	Solute carrier family 4 member 1	100514249	Transporter for anions across cell membranes
TRPV1	4.18	0.0132	Transient receptor potential vanilloid 1	100519212	Mainly involved in body temperature regulation, and pain sensing.

^1^Postive and negative FC represent up or down regulation, respectively.

As shown in Table 4, three pathways related to hematology were upregulated. Because all three have cross-over effects with each other in relation to blood coagulation, they will be discussed concurrently (Lei et al., 2013; Boknas et al., 2014; Troisi et al., 2021). First, the gene clustering associated with hematocrit was increased (*Z*-score = 1.360, *P* < 0.05). Hematocrit directly affects the ability of platelets to form a plug to stop bleeding (Ouaknine-Orlando et al., 1999; Kunicki et al., 2009). Second, the pathway responsible for production of red blood cell platelets was increased (*Z*-score = 0.827, *P* < 0.05). They are essential for hemostasis in that they aid in forming a red blood cell platelets plug and release co-factors that promote coagulation (Jackson, 2007).

Finally, hemostasis (*Z*-score = 0.848, *P* < 0.05) occurs because of complex mechanisms that lead to cessation of bleeding (Lei et al., 2013; Perrella et al., 2021). The combination of hematocrit and the upregulation of GP6 (2.68 FC, *P* = 0.0036, Table 5) work together to coordinate hemostasis (Kunicki et al., 2009; Perrella et al., 2021).

## Conclusions

In this experiment, we evaluated DGE and metabolic pathway analysis in neonatal piglets receiving one or two injections of iron dextran. RNA-seq was used to detect gene expression in the liver and duodenum of piglets at weaning. The results provide both novel observations as well as confirmation of known genes that are affected by iron status in neonatal piglets.

The results presented herein suggest that further study is needed to increase our understanding of the genes and pathways of hematological significance in older piglets and additional tissues such as marrow, spleen, and muscle. Furthermore, the results suggest that there are exciting relationships between Fe status of an animal and metabolic responses beyond the usually assessed hematological responses.

## Abbreviations

ADP: adenosine diphosphate
ATP: adenosine triphosphate
bp: base pair
BW: body weight
CON: control
DGE: differential gene expression
FC: fold change
+Fe: 2 iron injection treatment
Gpx-3: glutathione peroxidase 3
GTP: guanosine triphosphate
Hb: hemoglobin
IM: intramuscular
IDA: iron deficiency anemia
IPA: Ingenuity Pathway Analysis
mRNA: messenger ribonucleic acid
qRT-PCR: —quantitative real-time polymerase chain reaction
RNA: ribonucleic acid
RNA-seq: RNA sequencing
rRNA: ribosomal ribonucleic acid
Wt: weight
